# Genetic variation in a colonization specialist, *Simulium ruficorne* (Diptera: Simuliidae), the world’s most widely distributed black fly

**DOI:** 10.1371/journal.pone.0205137

**Published:** 2018-10-03

**Authors:** Mouna Cherairia, Peter H. Adler

**Affiliations:** 1 Laboratoire de Recherche et de Conservation des Zones Humides, Université 8 Mai 1945 de Guelma, Guelma, Algeria; 2 Department of Plant and Environmental Sciences, Clemson University, Clemson, South Carolina, United States of America; Fralin Life Science Institute, Virginia Tech, UNITED STATES

## Abstract

The ability of aquatic insects to colonize Earth’s most remote freshwater habitats, such as those of islands and deserts, is limited to select taxa. Among black flies, the premiere colonization specialist is *Simulium ruficorne* Macquart, the only species known from both the Afrotropical and Palearctic regions. We investigated the cytogenetics of *S*. *ruficorne* to gain insight into its wide geographic distribution and ability to colonize oceanic islands and deserts. On the basis of larval polytene chromosomes from 14 locations, we documented 17 novel and previously known chromosomal rearrangements and established five cytoforms (A1, A2, B, C, and D), of which probably four (A1/A2, B, C, and D) are distinct species and two (A1 and A2) represent sex-chromosome polymorphism involving a heteroband in the long arm of chromosome III. The chromosome restructuring phenomena associated with the five cytoforms are consistent with the trend in the Simuliidae that one and the same rearrangement can assume different functions in the various descendants of a common ancestor in which the rearrangement was polymorphic. The most widely distributed cytoforms are A1 and A2, which are found in North Africa, the Canary Islands, and Majorca. *Simulium ruficorne*, the only known black fly in the Hoggar Mountains of the central Sahara Desert, represents a cohesive population of cytoform A1 little differentiated from other North African populations of A1 and A2. Cytoform B inhabits the West African mainland, cytoform C is on Tenerife, and cytoform D is on Cape Verde. We suggest that dispersal and colonization specialists, such as *S*. *ruficorne*, are multivoltine inhabitants of temporary streams, and must relocate as their habitats deteriorate. *Simulium ruficorne*, therefore, should have adaptations that contribute to successful dispersal and colonization, perhaps largely physiological in nature, such as tolerance of high temperatures and droughts.

## Introduction

Islands are well represented among Earth’s biodiversity hotspots [1], with isolation contributing to the evolution of endemic forms [2]. Their biotic composition reflects the classic elements of island biogeography, such as island age, size, and distance from a source of potential colonists [3]. Insular biotas, consequently, are typically a mixture of endemic and widespread taxa.

Understanding the biotic composition and colonization history of islands depends on the clarity and precision with which taxa and their relationships are recognized. Island populations that appear structurally similar to mainland or other island populations can be endemic, cryptic members of a species complex [4,5]. Alternatively, populations that are structurally divergent, for example in color or size, can be conspecific with mainland populations, the differences reflecting founder effects and local selection [6]. Although determining the reproductive compatibility and taxonomic status of allopatric populations is challenging, genetic comparisons of island and mainland populations can provide clues to evolutionary relationships and the extent of divergence [7,8].

Although islands are conventionally viewed as oceanic entities, the island concept logically extends to ecological islands [9]. Desert mountains and oases are prominent examples of ecological islands. The Sahara Desert—the largest nonpolar arid region on Earth—has been a dispersal barrier promoting diversification of many organisms [10–12]. Its history is a complex mosaic of shifting climate and ecological conditions [13–16]. Embedded in a sea of Saharan sand are high-elevation areas, of which the Hoggar massif is the most prominent. It is of volcanic origin and consists of Precambrian formations rising to 2900 m above sea level [17,18].

Islands with flowing freshwater can provide habitat for aquatic organisms, such as black flies of the family Simuliidae. Black flies inhabit the vast majority of the world’s islands, attesting to their dispersal and colonization abilities [19]. Within the entire family, *Simulium ruficorne* Macquart is the premiere colonization specialist. It occurs throughout the Afrotropical and southwestern Palearctic Regions, including many of the associated islands, and is the only simuliid that occupies both zoogeographical regions [20]. It is also the only black fly that persists in the harsh environments of many isolated oases and massifs of arid regions such as the Sahara Desert and the Arabian Peninsula [21,22]. The presence of black flies in the mountains of the Sahara Desert has been known since the mid-1950s [23]. Subsequent surveys revealed that *S*. *ruficorne* is the only species known in the Saharan mountains [24,25].

Black flies, through studies of their polytene chromosomes, offer one of the richest databases of natural population genetics, and much of the taxonomy and systematics of the Simuliidae is now based on chromosomal characters [26]. The wealth of characters in the polytene complement has also been instrumental in discovering source areas of insular simuliids [27]. *Simulium ruficorne* presents an ideal case for genetic investigation because of its vast geographic range, presence in isolated areas such as islands, and morphological variation [22]. The genetic groundwork was laid by DG Bedo [28], who characterized the polytene chromosomes of four African populations of *S*. *ruficorne* and provided a standard chromosomal map for comparative biotaxonomic studies.

To gain insight into the wide distribution of *S*. *ruficorne* and its potential genetic variability, we characterized the banding patterns of its polytene chromosomes across multiple populations. Our focus was the diversity, divergence, and source of insular populations, including those of oceanic and desert islands.

## Materials and methods

### Ethics statement

Office National du Parc Culturel de l‘Ahaggar, Algeria, granted authorization to collect simuliids in the Hoggar Mountains, in accordance with provisions of Article 27 of Law 98–04 of 15 June 1998 on the protection of cultural heritage and of letter no. 015/DPLBCVPC/MC/16; 130/DPLBCVPC/MC/16. Collections in other countries were made on public land with access from public roads, and no permissions were required to collect material. No collections involved endangered or protected species.

### Collection and preparation of material

**Fig 1:**
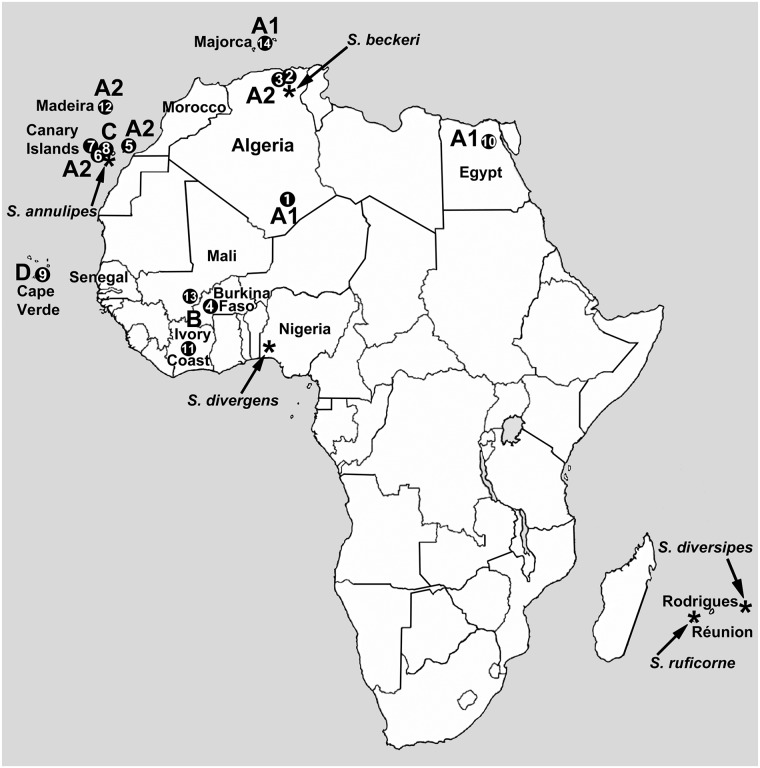
Map of Africa, showing all locations for which chromosomes of *Simulium ruficorne* have been analyzed. Numbers correspond with locations and data in Table 1. Type localities of *S*. *ruficorne* and its synonyms are indicated by arrows pointing to asterisks (*). Countries mentioned in the text are labeled, and cytoforms A1, A2, B, C, and D are associated with their collection localities. Source areas for colonization of the Canary Islands, Cape Verde, Madeira, Majorca, and the Hoggar Mountains are hypothesized to have been the nearest North African mainland areas.

**Fig 2:**
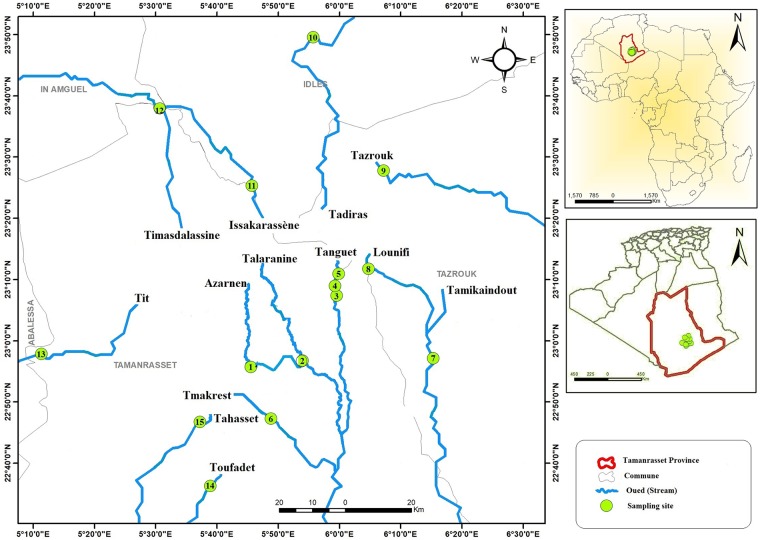
Map of collecting sites for *Simulium ruficorne* cytoform A1 in Hoggar Mountains, Algeria, November 2017. Maps were constructed with ArcGIS version 10.0.

**Fig 3:**
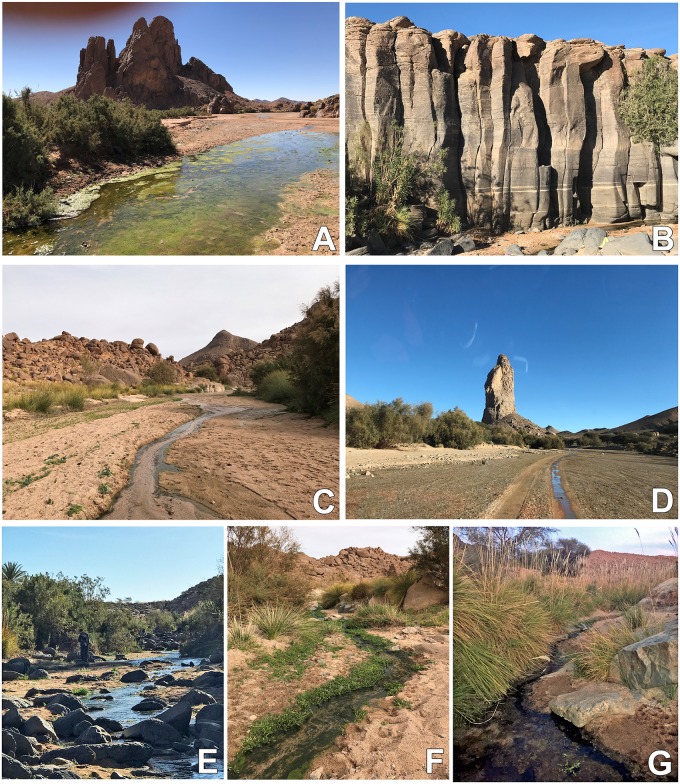
Examples of collecting sites for *Simulium ruficorne* cytoform A1 in Hoggar Mountains, Algeria, November 2017. A. Oued Tanguet in Tahifat, Titakaout. B. Oued Tadiras. C. Oued Lounifi. D. Oued Tanguet in Tahifat, Imaroues Mtn. E. Oued Tadiras, showing stream details. F. Oued Lounifi, showing stream details. G. Oued Tahasset.

Larval and pupal black flies were collected by hand from all available substrates in seven locations, and integrated with previously published cytological information from seven additional locations (Table 1; Fig 1). Intensive sampling was conducted at 15 sites in the Hoggar Mountains, Ahaggar National Park, in the Central Sahara of southern Algeria in November 2017 (Table 2; Figs 2 and 3). Larvae and pupae were fixed in 80% ethanol or in 1:3 acetic ethanol (Carnoy’s fixative). Representative specimens were deposited in the Clemson University Arthropod Collection, South Carolina, USA, except the Hoggar samples, which were deposited in the Laboratoire de Recherche et de Conservation des Zones Humides Collection, Université 8 Mai 1945 Guelma, Gulema, Algeria. Samples from the Hoggar Mountains were accompanied by the following physicochemical measurements of the streams: depth and width (meter stick and 10-m tape, respectively); current velocity (rate at which a cork moved 10 m); and water temperature, pH, conductivity, and salinity (portable multiparameter Hanna Instruments HI 9829 meter).

**Table 1:** Collection areas for chromosomal study of *Simulium ruficorne*.

Location No.	Location	Latitude	Longitude	Elevation (m)	Date of Collection
1	Algeria, Ahaggar National Park, Hoggar Mountains (15 sites; Table 2)	22°36′17″–23°49′32″N	05°11′19″–06°15′18″E	1132–1975	4–11 Nov 2017
2	Algeria, Souk Ahras Province, M’gisba^a^	36°04′29″N	07°29′40″E	747	29 Apr 2013, 15 Jan 2014
3	Algeria, Mila Province, Rejas	36°27′48″N	07°15′31″E	264	22 Oct 2016
4	Burkina Faso, Bouakan River^b^	—	—	—	10 Nov 1983
5	Canary Islands, Fuerteventura, near Vega de Rio Palmas^c^	28°24′N	14°05′W	265	23 Feb 2012
6	Canary Islands, La Gomera, Barranco de las Lagunetas	28°07′N	17°17′W	1100	13 May 2005
7	Canary Islands, La Palma, Barranco de las Angustias	28°42′N	17°55′W	635	24 Jun 2012
8	Canary Islands, Tenerife, Adeje (Barranco del Inferno), and Chio aqueduct^b^	28°08′–28°14′N	16°44′–16°47′W	500–800	7–8 Apr 1983
9	Cape Verde, Santiago Island^b^	—	—	—	4 Nov 1983
10	Egypt, Faiyum, valley of Wadi El-Rayan, streamlet Wadi El-Rayan^d^	29°08′52″N	30°23′33″E	-20	9 April 2015
11	Ivory Coast, Bouaké^b^	—	—	—	
12	Madeira, Faja Alta, trickle^c^	32°49′N	16°54′W	110	15 Mar 2011
13	Mali, Sikasso Region, Wolobougou	12°27′10″N	05°29′15″W	370	23 Aug 2017
14	Spain, Majorca, Torrent del Rec, Pollença	39°52′N	03°01′E	108	14 May 2014

^a^ Data from [29] (*n* = 3 larvae) and newly collected material (*n* = 14 larvae).

^b^ Data from [28]; details of collecting location not given.

^c^ Data from [30].

^d^ Data from [31].

**Table 2:** Sampling data for *Simulium ruficorne* in Hoggar Mountains, Tamanrasset Wilaya (Province), Sahara Desert, Algeria, 2017.

Region	Oued^1^	Latitude N Longitude E	Elevation (m)	Date	Larvae (*n*)	Pupae^2^ (*n*)	Chromosomes (♀:♂ larvae)
—	Azarnen	22°55’42” 05°45’33”	1655	4 Nov	66	1	7:4
—	Talaranine	22°56’42” 05°53’60”	1418	4 Nov	217	70	8:11
Tahifat, Imaroues Mtn.	Tanguet	23°07’30” 05°59’40”	1567	5 Nov	96	48	4:6
Tahifat, Ingriouel	Tanguet	23°08’56” 05°59’15”	1622	5 Nov	154	61	6:5
Tahifat, Titakaout	Tanguet	23°10’54” 05°59’55”	1672	5 Nov	233	28	6:4
Tamakerest	Tamakrest	22°47’14” 05°48’50”	1423	6 Nov	157	55	10:12
Tamikaïndout	Tamikaïndout	22°57’07” 06°15’18”	1238	7 Nov	79	2	7:9
Tazrouk	Lounifi	23°11’42” 06°04’44”	1917	8 Nov	300	69	13:6
Tazrouk	Tazrouk	23°27’45” 06°07’11”	1953	8 Nov	28	7	4:3
Idles	Tadiras	23°49’32” 05°55’41”	1415	9 Nov	220	63	8:3
Hirafouk	Issakarassène	23°25’19” 05°45’42”	1975	9 Nov	279	55	10:8
Hirafouk	Timasdalassine	23°38’02” 05°30’38”	1285	9 Nov	103	21	5:10
—	Tit	22°57’48” 05°11’19”	1132	10 Nov	159	67	5:6
—	Toufadet	22°36’17” 05°38’55”	1315	10 Nov	234	16	8:9
—	Tahasset	22°46’48” 05°37’11”	1432	11 Nov	40	11	6:7

^1^ Oued = stream; all flows were permanent except Tazrouk.

^2^ Pupae + exuviae.

The posterior portion of each larva was removed and stained with a modified Feulgen procedure [32]. Polytene chromosomes from larval silk glands and one gonad for gender determination were dissected out and squashed in 50% acetic acid under a coverslip on a microscope slide [33]. The banding sequences of our material were compared with the standard map [28] for *S*. *ruficorne s*. *l*. We photographed representative sequences under oil immersion on an Olympus BX40 compound microscope with a Jenoptik ProgRes^®^ SpeedXT Core 5 digital camera, and used Adobe^®^ PhotoShop^®^ Elements 8 to create chromosomal maps. All mapping conventions followed the precedent established for *Simulium ruficorne s*. *l*. [28]. However, we did not number the sections on our chromosome maps because the original standard map established for *S*. *ruficorne* [28] follows the section numbering for the *S*. *ornatipes* complex and, therefore, is substantially rearranged in many regions of the complement, creating a cumbersome and scrambled numbering system. New inversions were assigned numbers to follow the last number assigned for *S*. *ruficorne* in each arm [28]. All new inversions are precisely indicated on our maps by brackets, and each heteroband (hb) and band insertion (i) is indicated by an arrow. Fixed inversions are italicized on the maps and in the text. We identified the sex chromosomes when rearrangements could be linked to gender on one of the three chromosomes (I, II, or III) in either of the two arms, short (S) or long (L). If no rearrangements were linked to gender, we considered the sex chromosomes cytologically undifferentiated (X_0_Y_0_).

To interpret our results in a larger context, we integrated our findings with those of previously published chromosomal information on *S*. *ruficorne* [28–31]. The depiction of chromosomally defined relationships of cytoforms was based on the standard sequence established for *S*. *ruficorne s*. *l*. by Bedo [28], who chose a population from Cape Verde as the standard simply because it provided the best chromosomal preparations. The chromosomal tree is, therefore, unrooted and subject to revision once chromosomal sequences can be followed into appropriate outgroups [33] or molecular analysis of cytoforms provides a topology.

We presorted larvae from the Hoggar Mountains, for which we had a wealth of material, into two color categories: brown and dark gray. We also categorized all pupae and pupal exuviae from Algeria (Hoggar Mountains, M’gisba, and Rejas) according to previously established [22] gill configurations (‘A’ through ‘K’). In addition, we chromosomally analyzed larvae with different gill configurations in the Hoggar collections.

## Results

### Hoggar Mountain samples

Among 3146 larval and pupal simuliids collected from 15 sites in the Hoggar Mountains, *S*. *ruficorne* was the only species. All 15 Hoggar stream sites, except oued Tazrouk, were permanent, small (3–10 cm deep, 25–130 cm wide), cool to warm (17–27 °C), acidic (pH 6.4–6.7), and moderately slow (14–100 cm/sec), with low salinity (0.1–0.4%) and high conductivity (182–663 μS/ cm). Four additional stream sites were visited in the Hoggar Mountains, but had no black flies: oued Igherghir in Amadelnnanire (50 km from the border with Niger) and oued Igherghir (ca. 70 km from Tamanrasset) were dry, and oued Alrassoul (275 km from Tamanrasset) and oued Tamikaïndout (upstream of the collection site on the same stream) were reduced to stagnant pools.

Larval body pigmentation generally corresponded with the substrate to which the larvae were attached: brown if attached to vegetation and dark gray if attached to rocks.

Four pupal gill configurations were found in the Hoggar Mountains: ‘A-C’ (2.1%), ‘F’ (0.3%), ‘H’ (97.4%), and ‘K’ (0.2%). Gill forms ‘A’, ‘B’, and ‘C’ merged into one another in our material; we, therefore, combined these forms into one category (‘A-C’). ‘H’ was found at all Hoggar sites, whereas ‘A-C’ occurred at three sites (oued Tadiras, Issakarassène, and Tazrouk) and ‘F’ and ‘K’ at one site each (oued Tadiras and Tanguet, respectively). All 5 pupae from site 3 (Reja, Algeria) were ‘H’, and 17 of 18 pupae from site 2 (M’gisba) were ‘H’ and 1 was ‘A-C’.

### Chromosomal generalities

All populations of *S*. *ruficorne* had tightly paired chromosomal homologues and the nucleolar organizer in section 22 at the base of IL. The centromere bands of all larvae in all populations were monomorphically expressed as thick, darkly stained bands. Among 460 larvae, 17 rearrangements were detected, although 8 had a frequency of less than 0.05 (Table 3). Heteroband 83B (hb83B) was present as a single thick or thin band in all populations. IIL-1 also was present in all populations, although its status was unknown in Bedo’s [28] incompletely analyzed Burkina Faso population.

**Table 3:** Summary of chromosomal rearrangement frequencies in larvae of *Simulium ruficorne*.

Location	n ♀:♂^a^	IIS-1	IIL-1	IIL-2	IIL-3	IIL-6	IIL-7	IIIL-1	IIIL-2	IIIL-4	IIIL-6	hb83B	Cytoform	Reference
**Algeria**														
Hoggar Mts.	107:103		1.00				1.00	1.00	1.00	0.04		> 0.99	A1	Current Study
M’gisba	7:10		0.41				1.00	1.00	1.00			*^b^	A2	[29], current study
Rejas	8:5		0.27				1.00	1.00	1.00			**^c^	A2	Current study
**Burkina Faso**	6		?^d^	?	?	?	?					1.00	B	[28]
**Canary Islands**														
Fuerteventura	15:10		0.80				1.00	1.00	1.00			*^b^	A2	[30]
La Gomera	1:0		1.00				1.00	1.00	1.00			***^e^	A2	Current study
La Palma	10:6		1.00				1.00	1.00	1.00			****^f^	A2	Current study
Tenerife	73		0.98					1.00				0.76	C	[28]
**Cape Verde**	30	0.10	0.10	#^g^	0.03				0.03			0.88	D	[28]
**Egypt**^h^	0:2		0.50				1.00	1.00	1.00			0.25	A1	[31]
**Ivory Coast**	20		1.00			1.00		0.22				0.98	B	[28]
**Madeira**	1:4		0.90				1.00	1.00	1.00			****^f^	A2	[30]
**Mali**, Wolobougou	17:14		1.00			1.00					0.81^i^	0.97	B	Current study
**Spain** (Majorca)	6:5		0.64				1.00	1.00	1.00			0.64^j^	A1	Current Study

Note: The following rearrangements each had a frequency ≤ 0.02: IIS hc42 (< 0.01) in Tenerife [28]; IIL-4 (0.02) in Cape Verde [28]; and IIL-8, IIIL-5, IIIL-7, and IIIL hb86B1 (< 0.01) in Hoggar Mountains.

^a^ Larval gender was not reported for samples analyzed by Bedo [28], who gave only the total number of larvae examined.

^b^ * = The thick band was X-linked; 1 female sex exception was heterozygous for the thick band in each sample.

^c^ ** = The thick band was X-linked; 2 female sex exceptions were heterozygous for the thick band, and 1 male sex exception was homozygous for the thick band.

^d^ IIL could not be analyzed in a previous study [28]; therefore, the presence or absence of inversions IIL-1, IIL-2, IIL-3, IIL-6, and IIL-7 in the Burkina Faso population could not be determined, as indicated by question marks (?). We tentatively assigned the Burkina Faso population to cytoform B.

^e^ *** = The single female was homozygous for 83B; sex linkage could not be determined without males. We tentatively assigned the La Gomera population to cytoform A2.

^f^ **** = The thick band was X-linked; sex linkage was complete (no sex exceptions).

^g^ # = IIL-2 was partially Y-linked in the study by Bedo [28].

^h^ The sample had only 2 males, both heterozygous for IIL-1; sex linkage could not be determined without females and a larger sample. The population was tentatively assigned to cytoform A1.

^i^ Although females were predominantly inverted homozygotes (13 homozygotes vs. 4 heterozygotes) and males were predominantly heterozygotes (8 heterozygotes vs. 6 inverted homozygotes), IIIL-6 was not significantly sex-linked (χ^2^ with Yate’s correction = 0.163, df = 1, P = 0.6862).

^j^ Although the sample size was small, hb83B was in Hardy-Weinberg equilibrium (4 ++, 6 +-, 1 —, where ‘+’ = thick band and ‘-‘ = thin band; χ^2^ = 0.35, df = 1, P > 0.05).

Among all chromosomally known populations of *S*. *ruficorne*, we established 5 cytoforms (A1, A2, B, C, and D) on the basis of 7 rearrangements (IIL-1, IIL-2, *IIL-6*, *IIL-7*, IIIL-1, IIIL-2, and hb83B) that variously functioned as fixed, autosomal, or sex-linked (Tables 3 and 4).

**Table 4:** Sex chromosomes based on heteroband 83B in populations of *Simulium ruficorne* cytoform A2.

Site	Sex chromosomes			
	X_0_X_1_	X_1_X_1_	X_1_Y_0_	X_1_Y_1_
Algeria, M’gisba	1	6	10	
Algeria, Rejas	2	6	4	1
Fuerteventura	1	14	10	
La Gomera		1		
La Palma		10	6	
Madeira		1	4	

X_0_ and Y_0_ = thin band; X_1_ and Y_1_ = thick band.

### Cytoform A1

**Fig 4:**
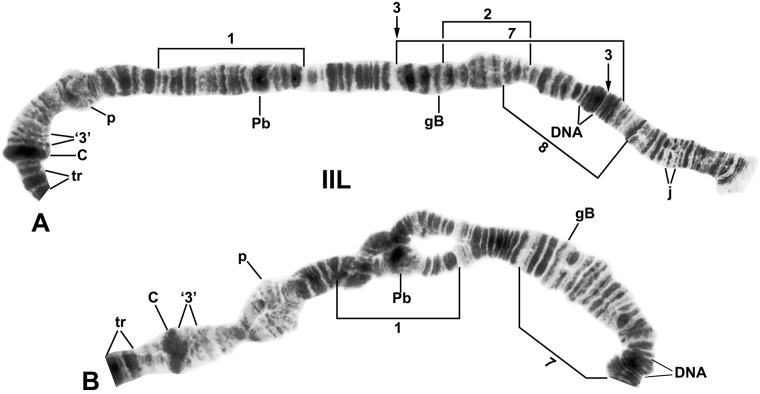
Chromosome arm IIL of *Simulium ruficorne* cytoform A1 (female larva). Landmarks and rearrangements are indicated by brackets and arrows. C, centromere; DNA, DNA puff; gB, gray band; j, jagged; p, puffing band; Pb, parabalbiani; tr, trapezoidal (in the base of IIS); ‘3’, 3 sharp. A. Entire arm showing the *IIL-1*, *IIL-7* sequence (female larva, Algeria, Hoggar Mountains, oued Tasrouk); breakpoints of IIL-2, IIL-3, and IIL-8 are indicated by brackets. B. Proximal majority of arm showing the *IIL-7* sequence with IIL-1 in heterozygous configuration (female larva, Majorca).

**Fig 5:**
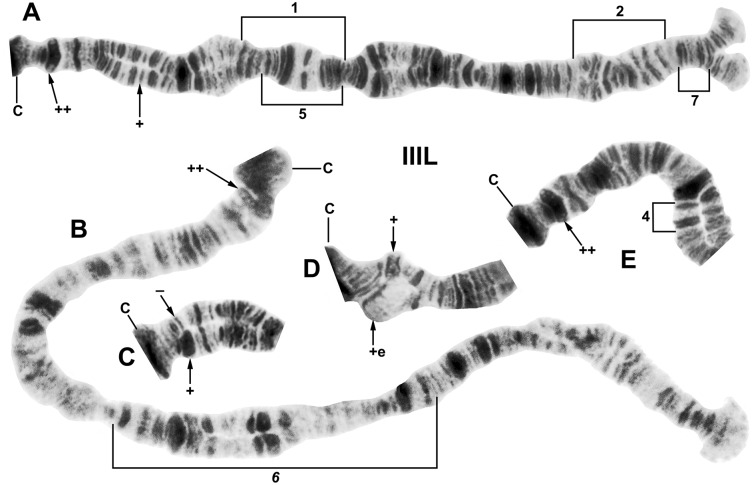
Chromosome arm IIIL of *Simulium ruficorne*. Landmarks and rearrangements are indicated by brackets and arrows; C, centromere. A. Entire arm of cytoform A1, showing the *IIIL-1*, *IIIL-2* sequence with hb83B expressed homozygously (++) (female larva, Majorca); breakpoints of IIIL-5 and IIIL-7 are indicated by brackets and location (+) of hb86B1 is shown with an arrow. B. Entire arm of cytoform B, showing the IIIL-6 sequence with hb83B homozygously expressed (++) (female larva, Mali, Wolobougou). C. Base of arm of cytoform A1 (male, Majorca), showing hb83B heterozygously expressed as a thin (-) and thick (+) band. D. Base of arm of cytoform A1 (male, Algeria, Hoggar Mountains, oued Tasrouk), showing hb83B as a thick band (upper homologue, +) and puffed (lower homologue, +e). E. Base of arm of cytoform A1, showing hb83B homozygously expressed (++) and IIIL-4 in heterozygous configuration; the lower homologue carries the inversion (male, Algeria, Hoggar Mountains, oued Issakarassène).

Samples from Algeria’s Hoggar Mountains and Majorca were fixed for *IIL-7* (Fig 4A), *IIIL-1*, and *IIIL-2* (Fig 5A) and had undifferentiated sex chromosomes (X_0_X_0_, X_0_Y_0_) (Table 3). IIL-1 was fixed in the Hoggar Mountains but polymorphic in Majorca. Heteroband 83B was autosomally polymorphic in Majorca but nearly fixed in the Hoggar Mountains, appearing heterozygously in 1 female and 1 male (Table 3). Consequently, the Hoggar and Majorca populations could represent separate cytoforms or ends of a cline. Given the geographic distance (ca. 1720 km) separating the two areas and the small Majorcan sample (*n* = 11), we assign them to the same cytoform. The two males from Egypt were tentatively assigned to cytoform A1; they were fixed for *IIL-7*, *IIIL-1*, and *IIIL-2*, and heterozygous for IIL-1, whereas one male was heterozygous for hb83B (as in Fig 5C) and the other was homozygous for the thin band. A single Hoggar male larva from Tazrouk stream was heterozygously puffed for hb83B in 90% of its nuclei, possibly representing gene expression (Fig 5D). Five rare polymorphisms were found in A1, but only in the Hoggar Mountains. IIL-8, IIIL-5, IIIL-7, and hb86B1 (Figs 4A and 5A) occurred as single heterozygotes, the first two in females, and the latter two in males. IIIL-4 (Fig 5E), although in low frequency (0.04), was heterozygous in males and females at 9 of 15 sites, providing additional evidence that *S*. *ruficorne* in the Hoggar Mountains is a single cohesive population. Ectopic pairing between centromere bands and between CI or CII and hb83B occurred in scattered nuclei of at least some larvae in all populations, suggesting some structural or functional genomic significance; ectopic pairing of telomeres was infrequent (< 5% of nuclei per population).

### Cytoform A2

Cytoform A2 from northern Algeria, the Canary Islands (Fuerteventura, La Palma, and possibly Gomera), and Madeira differed from A1 only by having differentiated sex chromosomes; hb83B was linked to the X chromosome (X_1_X_1_, X_1_Y_0_), although with about 6% sex exceptions (Tables 3 and 4). Thus, cytoforms A1 and A2 were distinguishable only at a population level. Other than IIL-1, autosomal polymorphisms were absent. Ectopic pairing was similar to that observed in cytoform A1.

### Cytoform B

Samples from the West African mainland (Ivory Coast, Mali, and possibly Burkina Faso) were fixed for *IIL-1* and *IIL-6* (Fig 4) and fixed or nearly so for hb83B (Fig 5). They lacked IIL-7 and IIIL-2, carried IIIL-1 as an autosomal polymorphism, and had undifferentiated sex chromosomes (X_0_X_0_, X_0_Y_0_) (Table 3). The high frequency (0.81) of IIIL-6 (Fig 5B), with slight but nonsignificant tendency toward X-linkage in the Mali population (Table 3), was evidence of additional differentiation within cytoform B. Although Bedo’s [28] analysis of the Burkina Faso sample was incomplete, we assigned it to cytoform B based on the absence of IIIL-1 and IIIL-2. Other than IIIL-1, IIIL-6, and hb83B, cytoform B lacked autosomal polymorphisms. Ectopic pairing of centromeres was observed in some nuclei of about 10% of Mali larvae, and the telomere band of IIIS was often slightly enhanced.

### Cytoform C

We assigned 2 populations analyzed by Bedo [28] from Tenerife (Canary Islands) to cytoform C. The populations were nearly fixed for IIL-1, lacked IIL-7 and IIIL-2, and had a high frequency of autosomal heteroband 83B (0.76) and undifferentiated sex chromosomes (X_0_X_0_, X_0_Y_0_) (Table 3). Bedo [28] recorded 1 individual heterozygous for a heterochromatic insert (hc42) in IIS.

### Cytoform D

We established cytoform D to accommodate the Cape Verde population analyzed and described by Bedo [28]. It had no fixed inversions, lacked IIIL-1, carried IIL-1 and IIIL-2 in low frequency (≤ 0.10) and hb83B in high frequency (0.88), and had unique sex chromosomes based on partial Y-linkage of IIL-2 (Table 3). Bedo [28] indicated that hb83B was in Hardy-Weinberg equilibrium as an autosomal polymorphism, and that IIS-1, IIL-3, and IIL-4 were additional, low-frequency (≤ 0.10) autosomal polymorphisms.

### Relationships of cytoforms

**Fig 6:**
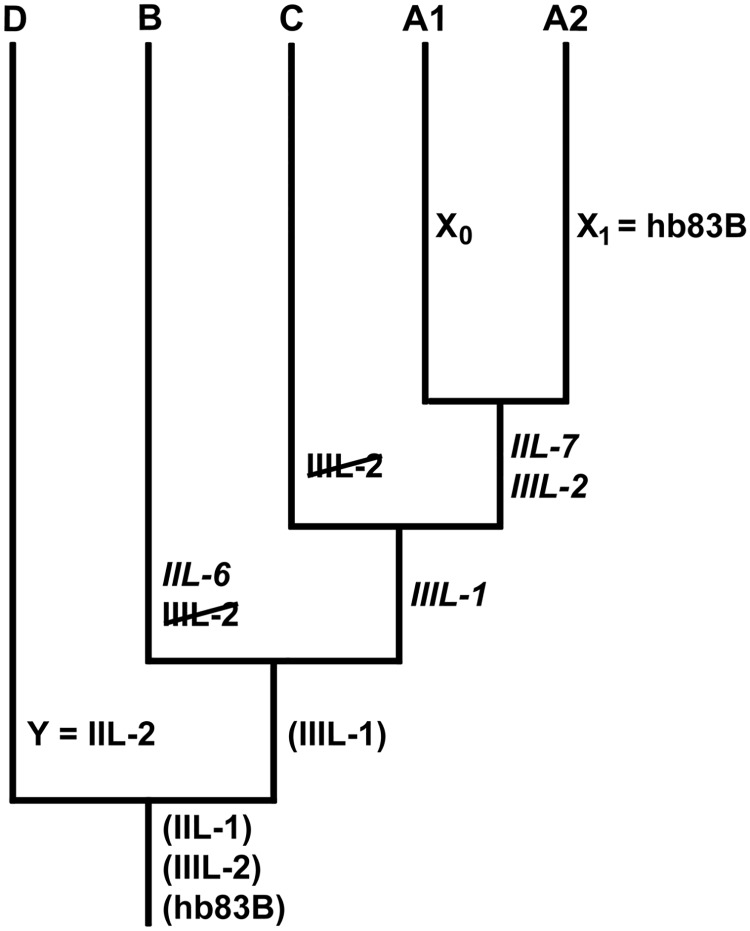
Chromosomal characteristics and relationships of five cytoforms of *Simulium ruficorne* expressed in an unrooted cytodendrogram. Parenthetical rearrangements are polymorphic, italicized inversions are fixed, inversions with a slash are lost, and sex chromosomes are indicated as X and Y; where sex chromosomes are not indicated, the X and Y are undifferentiated. Rearrangements shown at one node carry through to subordinate taxa, unless otherwise indicated.

In the unrooted cytodendrogram, cytoform D represented the standard sequence. Thus, it was the most similar cytoform to the hypothetical ancestor, which we show as polymorphic for IIL-1, IIIL-2, and hb83B (Fig 6). IIIL-1 is represented as a polymorphism in the ancestor of the remaining four cytoforms and is shown as retained as a polymorphism in cytoform B and fixed in the A and C cytoforms. IIIL-2 was retained as a polymorphism in D, fixed in A1 and A2, and lost in B and C.

## Discussion

### Taxonomic status of cytoforms

Broad geographic distributions often signal the presence of cryptic biodiversity in the Simuliidae [34,35]. *Simulium ruficorne* has the largest geographic range of any simuliid in the world [20], and has been a prime candidate for hidden diversity, now confirmed by the discovery of chromosomally distinct segregates. But which, if any, of the five cytoforms represent distinct species? The gold standard for testing species status of cytoforms—evidence of reproductive isolation in sympatry [36]—did not present itself in our study. We, therefore, offer hypotheses about the taxonomic status of the five cytoforms based on alternative evidence [37].

Sex-chromosome polymorphism is common in species of the Simuliidae and can involve either or both the X and Y chromosomes [38,39]. Cytoform D provides an example of sex-chromosome polymorphism in *S*. *ruficorne*; the sex chromosomes are either undifferentiated (X_0_Y_0_) or IIL-2 is linked predominantly to the Y chromosome (Y_1_) and infrequently to the X chromosome (X_1_) [28]. The occurrence of sex exceptions in some A2 populations might signify the presence of both X_0_ and X_1_ chromosomes. Cytoforms A1 and A2, therefore, possibly represent X-chromosome polymorphism in a single species. Pupal gill structure provides no discrimination between A1 and A2. Gill configuration ‘H’ is equally predominant in pupae from the chromosomally cohesive Hoggar Mountain population (A1) and Algeria’s Reja and M’gisba populations (A2). Differences in larval body pigmentation are related to the substrate to which larvae attach, a phenomenon also found in other simuliids [40]. We, therefore, tentatively regard A1 and A2 as merely cytotypes of a single species.

The consistency of the fixed chromosomal sequence specific to cytoform B over a distance of at least 500 km and the association with ecoregions distinct from those of all other cytoforms argue for species status. All three populations of cytoform B lie in the West Sudanian savanna and adjacent Guinean forest-savanna ecoregions [41]. Members of species complexes often differentially align with ecoregions, each of which presents a specific set of ecological attributes including stream conditions [42].

The presence of a pure population of cytoform C at two locations on Tenerife [28,43] among populations of A2 on the surrounding Canary Islands provides a compelling case for distinct species status. Cytoforms A2 and C are as near to one another as 55 km with about 28 km of the distance being ocean. The distance is well within the range of dispersal across open water for many simuliids [44], yet no indication of a mixed cytoform population has been found in the Canary Islands. We note, however, that *S*. *ruficorne* is common on Tenerife [43], and future collections might reveal the presence of A2, thereby providing a stronger test of reproductive isolation.

Cytoform D is the most geographically remote of our cytoforms. Unless it is found on the mainland, a decision about its specific status is stymied by the allopatric nature of being a distant island population. Cytoform D on Santiago Island (Cape Verde) is at least 700 km from the nearest mainland population, which is in westernmost Senegal [20]. Our analyses of *S*. *ruficorne* confirm the original observation [30] that inversion IIL-7 is similar to IIL-3, the latter of which is found in cytoform D [28], but IIL-7 includes four additional distal bands. We allow the possibility that IIL-3 is equivalent to IIL-7, given that the original analysis [28] involved only two IIL-3 heterozygotes with which to resolve the precise breakpoints. If IIL-3 is the same inversion as IIL-7, the chromosomal distinction between cytoforms D and A1/A2 decreases slightly.

Of the 17 total rearrangements known in *S*. *ruficorne*, seven (41%) are of value in establishing the cytoforms, and at least three of these seven (IIIL-1, IIIL-2, and hb83B) have different functions (fixation, autosomal polymorphism, loss, or sex-linkage) in the various cytoforms. Thus, the chromosomal restructuring of the cytoforms conforms to the evolutionary trend in the Simuliidae whereby a single rearrangement can assume different roles in different lineages arising from a common ancestor [45–47].

We suspect that *S*. *ruficorne sensu stricto*, with its type locality on Réunion (Fig 1), will be chromosomally distinct from all populations in our study, given the vast intervening geographical distance (> 6000 km). This hypothesis finds some support in a molecular study that places Moroccan and Réunion samples of *S*. *ruficorne* in separate clades [48]. *Simulium diversipes* Edwards, with its type locality about 825 km to the east of Réunion, on Rodrigues Island (Mauritius), could be conspecific with *S*. *ruficorne s*. *s*. If *S*. *ruficorne s*. *s*. has a restricted distribution (e.g., southern Africa and its islands), the widespread North African populations (cytoforms A1 and A2) would be *S*. *beckeri* Roubaud (type locality: Algeria), the Tenerife population (cytoform C) would be *S*. *annulipes* Becker, and the West African mainland populations (cytoform B) possibly *S*. *divergens* Pomeroy (type locality: Nigeria). If these assignments are accurate, only cytoform D on the Cape Verde Islands would lack a formal taxonomic assignment.

### Source areas of insular cytoforms

Seven samples of *S*. *ruficorne* are from oceanic islands and one set of 15 samples is from the Hoggar Mountains deep in the Sahara Desert. These samples provide an opportunity to examine dispersal and colonization, particularly source areas and chromosomal divergence. The A cytoforms of Madeira, Majorca, the Hoggar Mountains, and three of the Canary Islands probably represent colonizations from the nearest North African mainland areas, perhaps combined with island hopping in the Canaries. The chromosomal homogeneity of *S*. *ruficorne* across a wide swath of North Africa and adjacent islands suggests gene flow or recent colonization without sufficient time for divergence. Chromosomal uniformity over a wide area is perhaps expected, given the abundance and evident dispersal capabilities of *S*. *ruficorne* and its ability to colonize harsh lotic habitats, particularly in arid environments where it is found in the most remote oases and massifs of the Sahara [20]. Although *S*. *ruficorne* is principally ornithophilic [20,22], the probability that blood-feeding females have been transported to islands, such as Madeira, on birds is considered unlikely [49]. Rather, an argument has been made that *S*. *ruficorne* on Madeira is probably of wind-borne origin from North Africa, having arrived on the Leste, a dry easterly wind that blows from the Sahara over Madeira and the Canary Islands [30,49]. The chromosomal similarity of the Madeiran and most of the Canary flies with those of northern Algeria supports this hypothesis. If so, the chromosomal profile of *S*. *ruficorne* in Morocco, about 700 km from Madeira and 60 km from Fuerteventura (Canary Islands), should also match that of Algeria; that is, cytoform A2 should be found in Morocco.

The presence of a single cytoform (A1) of the only known simuliid morphospecies (*S*. *ruficorne*) in the Hoggar Mountains suggests that reaching the area, maintaining a presence, or both have been daunting. The Hoggar Mountains provide a reasonably stable thermal and humidity environment, similar to temperate areas, although typically with less rainfall [50]; our daytime measurements of stream temperatures were cool to warm (17–27 °C) and nearly all of the sampled flows that had simuliids are permanent. The streams, therefore, provide amenable thermal and flow conditions. A1 in the Hoggar Mountains probably colonized from northern areas. Colonization might have occurred via long-distance movements across inhospitable desert, perhaps from oasis to oasis, as suggested for *Drosophila* in North American deserts [51], or during the recent greening of the Sahara from 11,000 to 5,000 years before present [52]. Like cytoforms A1 and A2, the African green toad *Bufotes boulengeri boulengeri* (Lataste) shows weak genetic differentiation across North Africa, including geographically isolated populations in the Hoggar Mountains, a phenomenon attributed to distributional expansion during glacial periods; however, one haplotype is endemic to the Hoggar Mountains [12]. The fixation of IIL-1, near-fixation of hb83B, and unique presence of IIIL-4 in the Hoggar population of A1 indicates no successful incursion of cytoform B from the west and suggests that gene flow to and from the Hoggar Mountains is now limited. The Hoggar population has probably been isolated during the period of Saharan hyperaridity that persists today. We suspect that the chromosomally unstudied population in Tassili-n-Ajjer 300 km to the north of our nearest site in the Hoggar Mountains [24,53] is chromosomally similar to the Hoggar population.

The origins of cytoforms C and D on Tenerife and Cape Verde, respectively, are unclear. Either the cytoforms dispersed from respectively similar, contemporary mainland populations as yet undiscovered, or they diverged from their source populations during a period of extended insular isolation. Based on chromosomal relationships of the five known cytoforms, the latter hypothesis implies an origin of C from the ancestor of A1/A2 plus C. Fixation of *IIL-7* and *IIIL-1* in A2 but absence in C suggests that neither cytoform was derived from the other. Accordingly, cytoforms A2 and C on the Canary Islands would represent at least two separate, successful colonizations from the mainland. *Simulium tenerificum* Crosskey, a member of the *S*. *aureum* group, was considered an endemic species in the Canary Islands [43] until prospecting on the North African mainland revealed chromosomally identical populations, resulting in synonymy of *S*. *tenerificum* with *S*. *velutinum* (Santos Abreu) [54]. Thus, further collecting on the mainland might reveal the presence of cytoform C. Chromosomal relationships of cytoform D provide few insights into its origin. Collections from Senegal could be particularly informative.

### Dispersal and colonization attributes

We suggest that the premier dispersers and colonizers among the Simuliidae are multivoltine species that occupy temporary streams and must frequently move to other habitats as those they are using deteriorate. The relationship of dispersal and colonization to developing in temporary habitats holds not only for *S*. *ruficorne*, but also for other black flies, such as the continent-wide Nearctic *S*. *vittatum* complex, which can occupy temporary habitats and occurs on most North American islands [55,56], and *S*. *aureohirtum*, which colonizes temporary streams especially in disturbed (e.g., agricultural) areas throughout the Oriental Region into the Palearctic Region [57]. Given the chromosomal relationships of the cytoforms and the presence of four of the five cytoforms on oceanic and desert islands, the dispersal and colonization abilities of *S*. *ruficorne s*. *l*. must have been present in the ancestor of the cytoforms. The classic habitats of *S*. *ruficorne*—small, slow-flowing streams—are the original lotic habitat type on new volcanic islands and the final habitat type as islands age and erode [58], enhancing opportunities for colonization by *S*. *ruficorne*.

For aquatic insects generally, the relationship of structural characters to deteriorating habitats is classically expressed as dispersal polymorphism within and among species: apterous or brachypterous individuals occupy favorable habitats, whereas fully winged individuals occur in temporary or unstable habitats [59,60]. Little attention, however, has been paid to features of the Simuliidae, all of which are fully winged, which facilitate dispersal across uninhabitable areas, such as deserts and oceans. Dispersal has been correlated with a number of life-history traits in insects. Energetic and reproductive costs of dispersal, for example, decrease as body size increases in some *Drosophila* [61].

If wind is a significant factor in dispersal, as argued generally for simuliids [19] and particularly for *S*. *ruficorne* [43,49], then species given to routine dispersal might be expected to have a greater tendency to escape the boundary layer. The structural and behavioral correlates, however, are largely unexplored, although simuliids have been found 150 m or more above ground, and indirect evidence suggests that dispersing female flies can detect water and return to the boundary layer [19].

Parsing the role of dispersal ability separate from colonization ability in understanding distributions can be a challenge. Greater attention has been given to simuliid characters that favor colonization of marginally suitable habitats than to those that favor dispersal. Morphological attributes of simuliids that enhance fitness in harsh habitats include enhanced pigmentation of the larval nervous system, gonads, and head capsule to block ultraviolet radiation, toughened larval integument to reduce water loss, abdominal tubercles to increase surface area of the larval body for gas exchange, and an anterodorsal projection on the cocoon to stem water loss from the ecdysial line of pupae stranded above water [56]. These characters, however, are either not expressed in *S*. *ruficorne* or expressed no more so than in the majority of simuliids, suggesting that they are a preadaptation for colonizing harsh habitats or play little role.

Physiological mechanisms are probably of significance, such as those that enable eggs to survive periods of drought or permit larvae to tolerate low oxygen, high temperature, and high salinity. *Simulium ruficorne* is one of the most heat-tolerant simuliids on the planet, surviving in flowing waters of 35 °C [20]. Autogeny in the first generation of black flies has been suggested as an adaptive strategy for colonizing new habitats, although autogeny has not been found in *S*. *ruficorne* [62]. Higher reproductive potential should favor colonization of new habitats. Little is known, however, of fecundity and egg size in *S*. *ruficorne* [62]; studies of these fitness characters with regard to different cytoforms and their dispersal and colonization tendencies should prove fruitful.

High genetic diversity, as expressed in chromosomal polymorphism, is often associated with species that inhabit anthropogenically influenced streams and rivers [35,39]. Yet, *S*. *ruficorne s*. *l*. has relatively low levels of chromosomal polymorphism. Perhaps higher levels of genetic diversity exist at the molecular level. Exploring the genetic basis of characteristics associated with the foremost dispersers and colonizers, in concert with analyses of invasive species, could provide insights for predicting which species are likely to become invasive pests and vectors.
